# Direct observation of photocarrier electron dynamics in C_60_ films on graphite by time-resolved two-photon photoemission

**DOI:** 10.1038/srep35853

**Published:** 2016-10-24

**Authors:** Masahiro Shibuta, Kazuo Yamamoto, Tsutomu Ohta, Masato Nakaya, Toyoaki Eguchi, Atsushi Nakajima

**Affiliations:** 1 Keio Institute of Pure and Applied Science (KiPAS), Keio University, 3-14-1 Hiyoshi, Kohoku-ku, Yokohama 223-8522, Japan; 2 Department of Chemistry, Faculty of Science and Technology, Keio University, 3-14-1 Hiyoshi, Kohoku-ku, Yokohama 223-8522, Japan; 3 JST, ERATO, Nakajima Designer Nanocluster Assembly Project, 3-2-1 Sakado, Takatsu-ku, Kawasaki, 213-0012, Japan

## Abstract

Time-resolved two-photon photoemission (TR-2PPE) spectroscopy is employed to probe the electronic states of a C_60_ fullerene film formed on highly oriented pyrolytic graphite (HOPG), acting as a model two-dimensional (2D) material for multi-layered graphene. Owing to the in-plane sp^2^-hybridized nature of the HOPG, the TR-2PPE spectra reveal the energetics and dynamics of photocarriers in the C_60_ film: after hot excitons are nascently formed in C_60_ via intramolecular excitation by a pump photon, they dissociate into photocarriers of free electrons and the corresponding holes, and the electrons are subsequently detected by a probe photon as photoelectrons. The decay rate of photocarriers from the C_60_ film into the HOPG is evaluated to be 1.31 × 10^12^ s^−1^, suggesting a weak van der Waals interaction at the interface, where the photocarriers tentatively occupy the lowest unoccupied molecular orbital (LUMO) of C_60_. The photocarrier electron dynamics following the hot exciton dissociation in the organic thin films has not been realized for any metallic substrates exhibiting strong interactions with the overlayer. Furthermore, the thickness dependence of the electron lifetime in the LUMO reveals that the electron hopping rate in C_60_ layers is 3.3 ± 1.2 × 10^13^ s^−1^.

Research on the two-dimensional (2D) graphene material, which consists of a sp^2^ carbon network, has recently received a great deal of attention owing to its remarkable physical properties^1^,^2^, such as high electron conductivity and chemical stability, as well as optical transparency. While many practical possible usages of graphene have been proposed, a combination of graphene and an organic molecular film is of particular interest^3^,^4^,^5^,^6^,^7^,^8^,^9^,^10^,^11^,^12^,^13^,^14^, because they nicely suit the requirements for the next generation of low-cost, flexible, large area optical and electronic devices. From these viewpoints, an investigation of the electronic interaction and charge carrier dynamics at the graphene-organic interface is one of the key issues to resolve in the dawning era of graphene-based functional devices. Although the characteristics of organic molecules or functional nanomaterials deposited on a graphene surface have been investigated in this decade with scanning probe microscopy^4^,^6^,^8^,^9^,^12^, there have been few spectroscopic investigations relevant to the electronic structures and carrier dynamics at the hetero surface and interface.

Fullerene C_60_, consisting of a sp^2^ carbon network, is one of the materials most studied as a functional organic semiconductor since its discovery^15^ and it is also known to be a good electron acceptor for organic photovoltaic (OPV) devices, including solar cells, owing to its high electron affinity^16^,^17^,^18^,^19^,^20^,^21^. Indeed, graphene-C_60_ based OPV devices have been recently fabricated by several groups^3^,^7^,^11^,^14^. Since the transport gap (*E*_t_) of crystalline C_60_ is believed to be 2.3–2.4 eV^22^, it is likely that free photocarriers are generated with a photon energy (*hν*) considerably higher than 2.3–2.4 eV by the dissociation of highly-excited excitons (hot exciton). In fact, efficient photoconductance appears in a C_60_ film at *hν* > 2.3 eV^23^. So far, the ultrafast dynamics of photoexcited electronic states of C_60_ has been studied by several research groups^24^,^25^,^26^,^27^,^28^,^29^,^30^, but there are still insufficient spectroscopic investigation tracking precisely the photocarrier electron generated in C_60_ thin films.

In this article, we present a spectroscopic study of photocarrier electron dynamics of C_60_ deposited on a highly-oriented pyrolytic graphite (HOPG) as a model for multi-layered graphene by means of time-resolved two-photon photoemission (TR-2PPE) spectroscopy. The TR-2PPE unveils the ultrafast dynamics of electronically excited states on a surface system with femtosecond (fs = 10^−15^ s) time resolution^31^,^32^, and it has lately begun to be applied to graphene-related surfaces^33^,^34^.

The experimental results reveal that the energy levels of C_60_, which are independent of the C_60_ coverage, are little influenced by the 2D electronic nature of the in-plane sp^2^ carbon network; the overlayer molecular C_60_ film is electronically decoupled from the HOPG substrate. The photoexcited electron behavior in the lowest unoccupied molecular orbital (LUMO) of C_60_ suggests that free photocarrier electrons are generated by the dissociation of hot excitons without forming lower-lying excitons. The decay rate of photocarrier electrons into the HOPG substrate is very low (1.31 × 10^12^ s^−1^) compared with that on metallic substrates, which is also a characteristic of the 2D substrate. Moreover, thanks to the 2D nature of the material, it is revealed that the photocarrier electron hopping rate in C_60_ layers is 3.3 ± 1.2 × 10^13^ s^−1^, from the C_60_ thickness dependence of the TR-2PPE measurements.

## Results and Discussion

### Energy levels of C_60_ monolayer on HOPG

**Figure 1:**
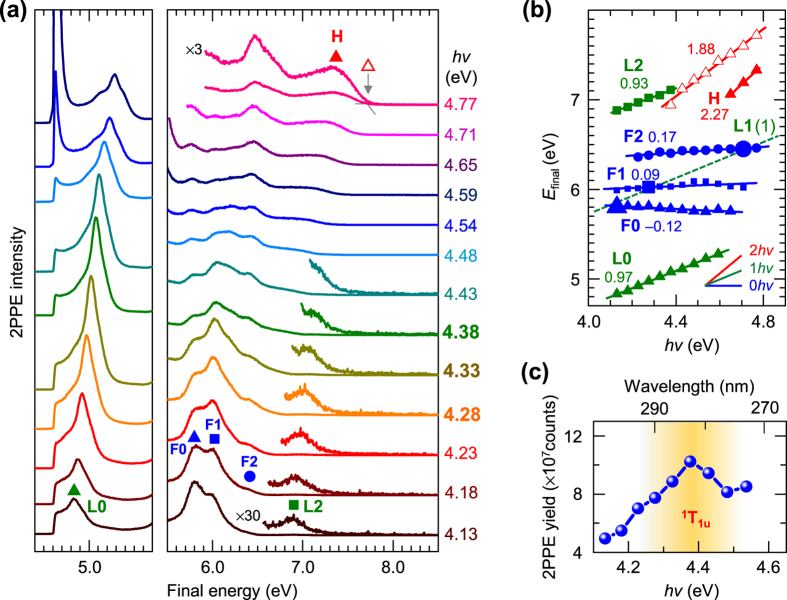
2PPE of 1 ML C_60_ on HOPG. (**a**) *hν* dependences of 2PPE spectra for 1 ML C_60_ film on HOPG aligned with *E*_final_, where the *hν* range was 4.13‒4.77 eV. (**b**) Energy positions of 2PPE structures including the onset of H against *hν*, which are assignable to the slope of “0 *hν*”, “1 *hν*”, or “2 *hν*”. Factors obtained by liner fits of the plots are indicated nearby the data. The dotted line shows the “1 *hν*” slope by an unoccupied level at *E*_F_ + 1.8 eV (see text). (**c**) Total 2PPE yield integrated whole energy region versus *hν* in which the maximum is corresponding to that optical absorption (^1^T_1u_) of C_60_ film. Note that recovering intensity at the highest *hν* is due to a contribution of one photon photoemission.

Figure 1a shows 2PPE spectra for a C_60_ monolayer (1 ML, see Supplementary Figs S1 and S2 for the coverage calibration) formed on HOPG by changing the incident *hν* at ~0.05 eV intervals. The photoelectrons emitted with surface normal (*k*_//_ = 0) were recorded with a limited acceptance angle of ± 0.6°. The horizontal axis shows the final state energy (*E*_final_) relative to Fermi level (*E*_F_). Six spectral features labeled as L0, L2, F0, F1, F2, and H appear by the formation of C_60_ film. The vacuum level (*E*_vac_) is located at 4.60 eV above *E*_F_ obtained from the energy of the low energy cut-off of the spectra with lower photon energy, while the intensity around the cut-off becomes stronger at *hν* ≥ *E*_vac_ because of signals from direct (one-photon) photoemission. When the peak positions in the 2PPE spectra, including a threshold of H (red open triangle), are plotted in Fig. 1b versus *hν*, they can be assigned to an initial, intermediate, or final state^35^; an occupied state below *E*_F_ (initial state: *E* = *E*_final_−2 *hν*), an unoccupied state between *E*_F_ and *E*_vac_ (intermediate state: *E* = *E*_final_−1 *hν*), or an unoccupied state above *E*_vac_ (final state: *E* = *E*_final_) are distinguished, whose shifts are proportional to 2 *hν*, 1 *hν*, and 0 *hν*, respectively. Note that the intensities of final states are enhanced at specific *hν* marked by larger symbols in Fig. 1b owing to a resonance with an intermediate unoccupied state at *E*_F_ + 1.8 eV, labeled by L1. In fact, the larger symbols are consistently fitted by a slope of 1 *hν* as a dotted line superimposed in Fig. 1b. Note that, the L1 structure appears more clearly in the 2PPE spectra with a wider acceptance angle (Supplementary Fig. S1) or in angle-resolved 2PPE (Supplementary Fig. S3), because it has an electronic resonance with dispersive F0–F2, as discussed below. Including the state at *E*_F_ + 1.8 eV, the 2PPE spectra unveil seven electronic states; one occupied state located at *E*_F_ − 2.2 eV (H), three unoccupied states between *E*_F_ and *E*_vac_ at *E*_F_ +0.7 (L0), +1.8 (L1), and +2.7 eV (L2), and three unoccupied states above *E*_vac_ at *E*_F_ +5.8 (F0), +6.0 (F1), and +6.5 eV (F2). Although the peak H (red solid triangle) is broadened below *hν* = 4.6 eV, which is assignable to an occupied state, judging from the plot of the slope aligning with 2 *hν*, including that of higher energy thresholds (red open triangle).

The series of F0–F2 states show large band dispersions parallel to the surface from an angle-resolved 2PPE measurement, while the others have little dispersion (see Supplementary Fig. S3). The result suggests that F0–F2 are assignable to a series of higher-lying superatomic molecular orbitals (SAMOs)^36^,^37^,^38^,^39^,^40^,^41^; the wavefunctions of SAMOs generally diffuse outside the molecular framework, which would form highly dispersive band structures in a molecular film with a periodic surface structure. As shown in Fig. 1a, F0–F2 are enhanced only around non-dispersive L1 (Supplementary Fig. S3), suggesting that the observation of SAMOs in 2PPE requires an initial or intermediate electronic state to allow the resonance. It is known that the lowest (1s-) SAMO is experimentally observed at around *E*_F_ + 3.3–3.8 eV in a C_60_ monolayer on noble metal substrates with a strongly delocalized nature^36^,^38^,^39^,^40^. The absence of the 1s-SAMO in our study is attributable to the 2D electronic structure of the HOPG substrate; since the electronic structures of graphene below and above *E*_F_ are governed by sp^2^ hybridized orbitals oriented parallel to the graphene plane, the chemical interaction with the overlayer material is very weak compared to metallic materials^8^. In fact, the energetics of a C_60_ film are almost independent (±0.1 eV) of the C_60_ coverage from 1 to 30 ML (see Supplementary Fig. S4), while metallic substrates considerably perturb the electronic states of C_60_, especially for the first layer, by strong metal-molecule coupling at the interface. Owing to the weak chemical interaction, an electron excitation through the conduction band of the substrate into the unoccupied states of C_60_ film would be unlikely. For C_60_ on HOPG, electron excitation into the 1s-SAMO is allowed only from an occupied level (e.g. the highest occupied molecular orbital (HOMO)) of C_60_ with intra- or inter-molecular excitation. Since the onset of the HOMO is *E*_F_–1.8 eV from the ultraviolet photoelectron spectroscopy (UPS) (Supplementary Fig. S5), the HOMO electron cannot access energetically to the 1s-SAMO with the current *hν*s (4 – 5 eV). Indeed, the 1s-SAMO observed in a 1 ML C_60_ film on Ag(111) with a similar *hν* quickly disappeared on additional C_60_ deposition^38^, indicating that injection of electrons from a metal substrate is essential to resolve the 1s-SAMO with the *hν* range used here.

More importantly, we now focus on the observed energy levels of H and L0–L2, which are essential to understand the electronic and optical properties of the C_60_-HOPG system, rather than higher-lying C_60_ SAMOs. Comparing with UPS result (see Supplementary Fig. S5), the peak H is assignable to HOMO of C_60_^22^,^42^.

For the assignments of L0, Dutton *et al*. had initially assigned it to LUMO of C_60_^28^, where they observed very similar 2PPE spectra with the present result for >2 ML C_60_ coverages of Cu(111) substrate. However, they subsequently reassigned the corresponding peak (L0) to the next LUMO (LUMO + 1) involved in Frenkel excitions^29^,^43^. The main reason of their change in the assignment relied on the energy matching with a theoretical result for the optical transition in solid C_60_^44^. They have also argued that the LUMO (exciton) is expected to be located at slightly above *E*_F_, which cannot be resolved using their experimental conditions. Another 2PPE group using a pump photon of 3 eV has insisted on other assignments of 2PPE structures; peaks observed at HOMO + 2.2 eV, and +3 eV are attributed to free photocarrier electron in LUMO, and LUMO + 1, respectively^26^,^27^. In spite of their previous (re)assignments, judging from our experimental findings on HOPG substrate, it should be more reasonable to assign L0 located at HOMO + 2.9 eV as a photocarrier electron in LUMO for the following reasons.

C_60_ has a HOMO (*h*_u_) and LUMO (*t*_1u_), with an optical gap (*E*_opt_) of 1.7–1.9 eV^45^,^46^,^47^. The UPS and inverse photoemission spectroscopy (IPES) for a C_60_ film have shown that the LUMO is observed at *E*_F_ + 0.6 eV, which provides the HOMO-LUMO gap (*ΔE*) of 2.35 eV^48^. The energy difference (*ΔE*−*E*_opt_) is attributed to on-site Coulomb energy (exciton binding energy), *U*.

In the 2PPE spectroscopy, occupied states in a cationic final state are probed similar to UPS, whereas unoccupied states reflect a neutral or cationic final state depending on the excitation processes. There are two photoemission pathways via an unoccupied state: (1) When electrons are supplied from a donor state with a pump photon, the molecule can be regarded as an anion, and then the electron occupying originally unoccupied states is emitted by a probe photon. A probe photon of 2−6 eV can emit the photoelectron mainly from molecular anions in the top layer owing to the limited escape depth of electrons. In fact, the 2PPE spectra for 1 ML C_60_/Au(111), which show a strong molecule-substrate interaction are markedly changed at 2 ML^29^, suggesting that the photoelectron escape depth is around or smaller than the thickness of one monolayer.

The other pathway of (2) is via an intra-molecular excitation, in which a photo-excited *e-h* pair is localized at a molecule, resulting in a larger excitonic interaction between the electron and hole. After the intra-molecular excitation, the electron is emitted with the probe photon, where there are two channels of photoelectrons (2a) from the exciton and (2b) from photocarrier electrons. The latter is generated by the hot exciton dissociation into a free electron and hole.

For the C_60_ in the outermost layer, furthermore, the number of surrounding C_60_ molecules is smaller than that inside the C_60_, and then the polarization energy to stabilize a charged molecule becomes smaller for the surface molecules. In other words, the less crowded surroundings result in the increase of *U*, and the Δ*E* of the outermost layer is larger than that in the bulk. This effect should be paid attention to when the values are compared among the results of UPS, IPES, and 2PPE spectroscopy.

In bulk C_60_, *ΔE* has been estimated to be 2.35 eV from the thresholds of UPS and IPES^49^, while *ΔE* is 2.9 eV in the 2PPE spectroscopy obtained from H and L0 peaks. Based on the assignment of L0, indeed, the successive L1 and L2 peaks are assignable to LUMO + 1 and LUMO + 2 with reasonable energy positions observed by IPES^22^,^42^. These consistent results show that L0 is assignable to the LUMO of a neutral C_60_, where the photoemission from L0 occurs via process (2b).

It is known that *ΔE* for a C_60_ monolayer on a metal substrate is small compared to that in the bulk C_60_, because the on-site charge is more effectively screened by the substrate than by a C_60_ film, resulting in a reduction of *U*^49^,^50^,^51^. As mentioned above, the energy positions are almost identical regardless of the C_60_ coverage on HOPG (Supplementary Figs S1 and S4), which shows that the screening effect from HOPG is much smaller than that from a metal substrate, due to its low density of states near *E*_F_. Furthermore, as discussed below, electrons in L0 have a long lifetime of several picoseconds, also indicating that L0 *does not* originate from the LUMO + 1 involved in Frenkel excitions, as pervious reassignments^29^,^43^, otherwise it would decay rapidly into lower-lying states within a few hundred fs^52^,^53^,^54^. Another 2PPE group has observed a peak at HOMO + 2.2 eV using a 3 eV pump photon, and they have assigned it to the free photocarrier electron in the LUMO^26^,^27^. However, we believe the assignment is not appropriate, because the peak to peak energy separation of 2.2 eV is too small to assign to a free photocarrier in the LUMO, considering that the *ΔE* of 2.3 eV defined by the respective onset edges in the UPS and IPES spectra.

**Figure 2:**
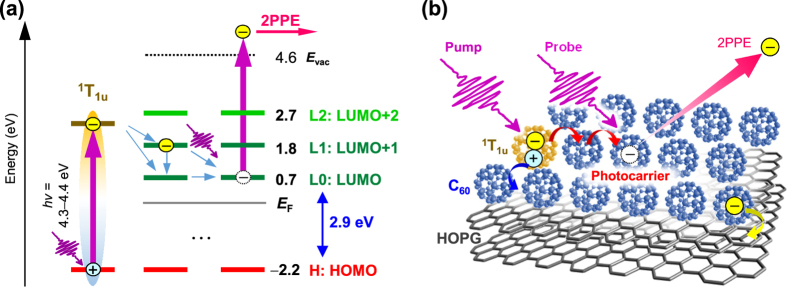
Energy diagram of observed energy levels. (**a**) Energy diagram of occupied and unoccupied levels near *E*_F_ as well as a hot exciton state of ^1^T_1u_. F0–F2 located above *E*_vac_ are not addressed in (**a**,**b**) Drawing of the hot exciton dissociation and hopping processes during the 2PPE.

**Figure 3:**
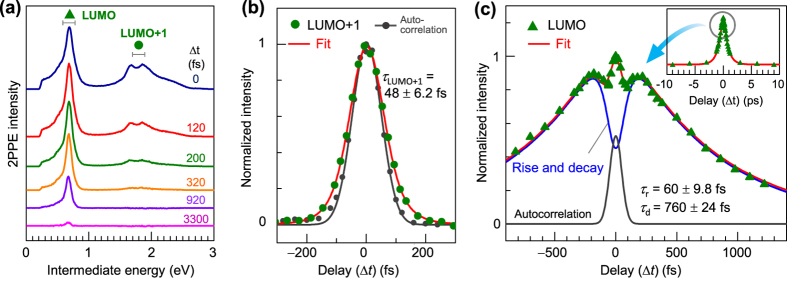
TR-2PPE spectra. (**a**) TR-2PPE spectra (*hν* = 4.33 eV) at various Δ*t*s. Horizontal axis stands for the intermediate energy defined as *E*_final_−1 *hν*. (**b**) Intensity trace of LUMO + 1 against Δ*t*, where the intensity is obtained from an integration of 0.1 eV width around *E*_F_ + 1.8 eV marked in **a**. The trace is fitted by a convolution of Gaussian autocorrelation (116 fs in FWHM) and single exponential decay functions. The autocorrelation function was determined in advance from the intensity trace of TR-2PPE for bare HOPG at around *E*_F_ + 1.5 eV (grey circle). (**c**) Intensity trace for LUMO. Fitted components of the first (Autocorrelation) and second terms (rise and decay) in eq. (1) are also overwritten as solid lines. Inset shows a wider Δ*t* region (±1  ps).

Despite the above, it seems reasonable that hot excitons are generated by pump photon in 2PPE spectroscopy. Figure 1c shows the *hν* dependence of the total 2PPE yield, which reaches a maximum at *hν* = 4.3–4.4 eV, implying a resonant photo-excitation in the C_60_ film. This energy indeed corresponds to the optical absorption of a C_60_ film (280 nm in wavelength)^23^,^55^, assignable to the third optically allowed resonance of 3^1^T_1u_^56^ marked in Fig. 1c. As seen in Fig. 1a, the intensity of LUMO + 2 at *E*_F_ + 2.7 eV is much weaker than that of the LUMO and LUMO + 1, also implying that electrons are dominantly excited from the HOMO at *E*_F_−2.2 eV with current *hν* of 4.8 eV at most; while the contribution of occupied states in HOPG is rather small. Therefore, it can be considered that hot excitons are nascently generated in the C_60_ film by a pump photon and subsequently dissociate into a free electron and hole without relaxing into lower-lying excitons. The excited electron temporally occupies LUMOs of surrounding non-excited C_60_, and is finally detected as a photoelectron excited by a probe photon. One may wonder why the hot exciton (3^1^T_1u_) is not resolved as an individual peak in the 2PPE spectra. The absence of the initially-excited hot exciton in 2PPE spectra would imply a very fast (<sub 10 fs) process of hot exciton dissociation, which broadens the spectral feature of the excitonic state (lifetime broadening)^32^. The observed energy levels and the excitation and detection scheme in 2PPE process are schematically illustrated in Fig. 2a,b. Here, it is not exclusive for a charge transfer (CT) state of C_60_^+^C_60_^−^ to be involved, and the CT state is actually predicted to be located at HOMO + 2.3 eV in theoretical calculations^57^. Formation of the lowest-lying excitons such as S_1_ and T_1_ would be another pathway of hot electron relaxation that may induce the photoemission by process 2a described above. The central energy of S_1_ (0-0 transition) is known to be 2.0 eV, whereas the absorption band of S_1_ distributes from 430 to 670 nm in wavelength^58^. Taking into account the energy of the HOMO (*E*_F_−2.2 eV), the higher energy component of S_1_ is expected to appear around the low energy cut-off (*E*_F_ + 0−0.7 eV in the intermediate energy (*E*_final_−*hν*)) and also to exhibit relaxation dynamics on the ps-ns time scale. However, such a spectroscopic feature in the low energy region and the dynamics could not be observed (See Figs 1a and 3a shown below), showing that the formation of lower-lying excitonic states is a minor process in the current *hν* range employed of 4–5 eV. The formation of S_1_ and T_1_ has been actually reported in 2PPE with different experimental conditions^26^,^27^,^30^; a 3 eV pump photon results in a lower efficiency of photocarrier generation than that with *hν* ~ 4.5 eV. Furthermore, they employed a higher laser intensity (~sub-μJ/pulse)^26^ with low repetition rates (250 kHz) and measured for thicker C_60_ film (20 nm ≈ 25 ML), which may favorably induce excitons by paring the free photocarriers in C_60_ film due to a high photoexcitation density. It should be emphasized again that the enormous intensity around the cut-off with *hν* > *E*_vac_ in Fig. 1a must be due to direct one photon photoemission from just below *E*_F_.

### TR-2PPE results

Figure 3a shows TR-2PPE spectra taken with various pump-probe delays (Δ*t*), where a spectrum at Δ*t* = 9 ps is subtracted as a background. The LUMO peak survives even at Δ*t* = 3 ps, while LUMO + 1 decays more quickly within a few hundred fs. The energy positions of these peaks remain almost the same, which again suggests that free electron photocarriers are generated in the early regime. Otherwise, they would subsequently form lower-lying excitons, (e.g. S_1_ or T_1_), which recombine. Figure 3b shows the intensity of LUMO + 1 against Δ*t*. The electron lifetime in the level is 48 ± 6 fs, evaluated by a convolution of a Gaussian autocorrelation (AC) (116 fs in full width at half maximum (FWHM)) and a single exponential decay, where the AC is determined from the intensity trace of the TR-2PPE for bare HOPG at the energy of *E*_F_ + 1.2–1.7 eV; the lifetime of the electron in the intermediate energy is very short (<20 fs)^59^ as compared to the true AC width. As shown in Fig. 3c, interestingly, the signal intensity for LUMO is once again recovered around Δ*t* = 100–250 fs after the excitation and gradually decreases. Assuming exponential rise and decay processes with time constants of *τ*_r_ and *τ*_d_, the time evolution of 2PPE intensity, *I*(Δ*t*), can be described as


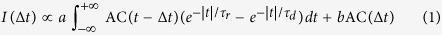


where *a* and *b* are weight factors. In addition to the rise and decay processes of LUMO electrons, the second term of the AC component is taken into account, because photoelectrons at the corresponding energy may include two components of (i) coherent 2PPE from occupied states of HOPG and HOMO−1 of C_60_ (see Supplementary Fig. S5) and (ii) short-lived hot electrons in the HOPG and C_60_ film, both of which are not regarded as the photocarrier electrons in the LUMO. The TR-2PPE trace for these components should be very similar to the shape of the true AC for the current pulse duration. Eq. (1) reproduces the evolution of the LUMO with *τ*_r,1ML_ = 60 ± 9.8 fs and *τ*_d,1ML_ = 760 ± 24 fs. Note that the rise time *τ*_r,1ML_ is close to the lifetime of LUMO + 1 electron (48 ± 6 fs), suggesting that the LUMO electrons are mainly supplied from highly excited states via LUMO + 1. The LUMO lifetime on HOPG is significantly longer than those for C_60_ on metal substrates^29^,^43^, e.g. Dutton and co-workers have reported that the lifetime increases with the C_60_ thickness on Au(111) from 79 fs at 2 ML to 340 fs at 30 ML, while the corresponding peak has been assigned to a LUMO + 1* exciton^29^,^43^. Since 2D material substrate of HOPG exhibits weak electronic coupling toward adsorbed species, the decay rate into HOPG is less compared to metal substrates. In fact, the energy positions in the 2PPE spectra for C_60_ films on HOPG show little changes with the C_60_ coverage (Supplementary Figs S1 and S4).

One should also be concerned about the effect of the excitation density on the TR-2PPE results; the recombination of photocarriers generated at different C_60_ molecules (bimolecular recombination) would contribute to the relaxation dynamics. However, the effect is negligible because of the sufficiently low excitation density used in the current study. In the TR-2PPE measurement, assuming a photon flux of ~3 × 10^10^ photons·cm^−2^ at most (76 MHz, 10 mW, and 0.1 mm spot diameter), three photons are irradiated in an area of 10 × 10 nm^2^ area per pulse (an area consisting of about a hundred of C_60_ molecules). Therefore, the excitation density would be too low to cause bimolecular recombination.

### Thickness dependence of LUMO lifetime

**Figure 4:**
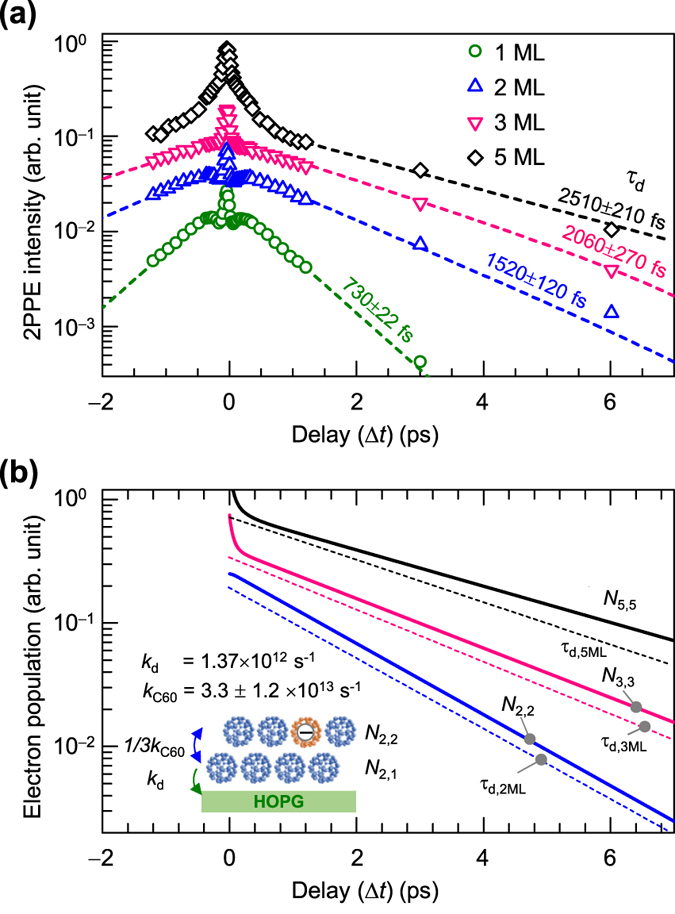
Thickness dependence of LUMO lifetime. (**a**) Intensity of LUMO against Δ*t* for 1, 2, 3, and 5 ML C_60_. Decay times are fitted using eq. (1) for 1–3 ML, while that for 5 ML is estimated by a slope at Δ*t* > 1 ps (dotted lines). (**b**) Simulated results with the interlayer hopping model for 2 ML (blue line), 3 ML (pink line), and 5 ML (black line). *τ*_d_ obtained in **a** are also shown as exp (−*t*/*τ*_d_) (dotted lines) to compare with the simulation (solid lines).

Since the sp^2^ hybridized nature of the HOPG substrate reduces photocarrier relaxation, the photocarrier hopping rate in a C_60_ film can be evaluated by the C_60_ thickness dependence of the LUMO lifetime. Figure 4a shows the time evolution of LUMO intensities obtained for various coverages of C_60_ films. The recovery of LUMO intensity around Δ*t* = 100–250 fs becomes unclear at ≥2 ML, while the lifetime of LUMO + 1 is independent of the coverage (see Supplementary Fig. S6). As discussed in the previous section for spectral assignments, 2PPE probes the photocarrier electrons remaining in the topmost C_60_ layer even for the multilayered films. On the other hand, the photocarrier electrons are generated by the pump photon penetrating into whole C_60_ layers. When the photocarrier electrons at the topmost layer initially originate not only from the topmost layer but also at the deeper layers inside, the rise time behavior should become vaguer in the multilayer films, as seen in Fig. 4a. The result therefore indicates that the created photocarriers move around in the C_60_ film with a finite hopping rate. To evaluate the photocarrier hopping rate in the C_60_ film, the thickness dependence of the decay phenomena was modelled for the LUMO electrons.

As shown in Fig. 4a, the LUMO lifetime lengthens with the thickness, and the fitting with eq. (1) shows that *τ*_d_ for 1, 2, 3, and 5 ML are 730 ± 22, 1520 ± 120, 2060 ± 270, and 2510 ± 210 fs, respectively. With increases in the C_60_ thickness, a uniform layer-by-layer structure grows without the presence of non-uniform islands (see Supplementary Fig. S7). Since the lifetime of a free electron carrier in solid C_60_ is much longer (ns or *μ*s) than the observed *τ*_d_^60^, the decay of photocarriers mainly occurs at the first C_60_ layer interfaced with the substrate. When the photocarrier electron hopping rate from an original C_60_ site to the nearest neighboring C_60_ is defined as *k*_C60_, the rate to the specific neighboring C_60_ should be 1/12 *k*_C60_, because of the fcc crystal structure of a C_60_ film exhibiting a coordination number of twelve. Considering that the fcc’s C_60_(111) surface faces to the vacuum, the interlayer hopping rate from original C_60_ site at the *i* th layer to the *i* + 1 or *i* − 1 th layer is 3/12 *k*_C60_ each (six neighboring C_60_ molecules exist at the *i* th layer). In addition, it can be assumed that the interlayer hopping from top and bottom layers are modified to be 3/9 *k*_C60_ owing to the absence of a layer to one side. Based on these concepts, here, the electron population of each layer, *N*_*n,m*_, is evaluated, in which *n* is the total number of C_60_ layers, and *m* is the targeted C_60_ layer (1 ≤ *m* ≤ *n*). First, we simply evaluate the value of *k*_C60_ for the result for 2 ML: the number of LUMO electrons at the first interface layer (*N*_2,1_) and the top layer (*N*_2,2_) can be simply described by


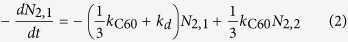



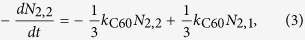


where *k*_d_ is the decay rate of the photocarrier electron density by relaxing into the substrate from the first C_60_ monolayer, i.e. *k*_d_ = 1/*τ*_d,1ML_ = 1.37 × 10^12^ s^−1^. Since the irradiated photon penetrates through the C_60_ films, it is reasonable to assume that all of C_60_ layers are uniformly photoexcited as *N*_2,1_ = *N*_2,2_ = 1 at *t* = 0, whereas the time trace of the TR-2PPE reflects the photocarrier at the top layer (*N*_2,2_). Then, the above differential equations can be solved numerically for the variable *k*_C60_. The blue solid line in Fig. 4b is the simulated result for *N*_2,2_, where *k*_C60_ is evaluated to be 3.3 ± 1.2 × 10^13^ s^−1^. So far, the electron hopping rate in unoccupied energy levels has been evaluated by analysis of the resonant autoionization spectrum which has been established as core-hole-clock-spectroscopy^61^. In this method, however, the accessible frequency range of 10^16^ > *k* > 10^14^ s^−1^ is limited by the lifetime of the core hole (~a few fs). In fact, Brühwiler *et al*. have evaluated the hopping rate of a LUMO + 1 electron in solid C_60_ to be ~1.7 × 10^14^ s^−1^, while they could not evaluate it for the LUMO^62^. The difference in the hopping rates depending on the energy levels can be explained by the width of the density of states^63^; the hopping rate would become higher for a wider band width. From the resonance window of LUMO + 1 (~0.5 eV) with SAMOs (supplemental material, S3), the band width is larger than that of LUMO (~0.2 eV in FWHM).

The above simulation can also be applied to the result for 3 ML. By using the central value, 3.3 × 10^13^ s^−1^, for *k*_C60_ as obtained above, however, the simulation for 3 ML slightly overestimates the experimental data (not shown). This could be caused by the polarization effect; owing to the reduction of *U* at the topmost C_60_, it can be qualitatively deduced that the hopping rate of electrons from an interior C_60_ layer to the topmost layer should be smaller than that from the topmost to an interior layer. Indeed, the result for 3 ML could be well simulated when the former and latter rates are modified to be 2.1 × 10^13^ and 4.5 × 10^13^ s^−1^ (pink solid line in Fig. 4b), respectively, as upper and lower limits of the error for *k*_C60_ (±1.2 × 10^13^ s^−1^). The reasonable modification falling within its uncertainty suggests the validity of our interlayer hopping model where hopping within an in-plane intralayer does not contribute explicitly. Note that the simulation for 5 ML (*N*_5,5_) overestimates the experimental result (*τ*_d,5ML_), possibly due to thickness inhomogeneity (see Supplementary Fig. S7 for scanning tunneling microscopy images) that could open a decay channel through an edge state of C_60_ layers.

We think that the hopping rate obtained with the above analysis is intrinsic for organic species, but independent of the substrate. The analysis will therefore be also applicable universally for the determination of *k*_d_ at the interface between an organic film and a metallic substrate, where *k*_d_ is typically too large to be resolved experimentally because of the strong substrate-molecule interaction.

## Summary

We observed the hot exciton dissociation and decay dynamics of generated photocarrier electrons in a C_60_ film on a HOPG substrate through the use of TR-2PPE spectroscopy. Unlike metal substrates, the 2D material substrate of HOPG substrate suppresses the electronic interaction with overlayered C_60_ film, which enables us to detect clear time required for photocarrier generation within a hundred fs. The time evolutions of the 2PPE intensity and their C_60_ thickness dependence reveal that the generated photocarriers randomly and frequently hop in the film until decaying into the substrate, where the electron hopping rate in C_60_ is independently and directly evaluated to be 3.3 ± 1.2 × 10^13^ s^−1^. The results have clarified fundamental aspects of photocarrier generation and carrier transportation in organic materials with time-domain measurements, and will provide a key to improving the performance of graphene-based organic devices.

## Methods

### Sample preparation and 2PPE measurements

Prior to C_60_ deposition, an HOPG substrate was cleaved in air and cleaned by heating (670 K, 50 h) in an ultrahigh vacuum (UHV) chamber, where the base pressure was better than 1 × 10^−8^ Pa. C_60_ was then deposited in the UHV system with a rate of 0.07 ML/min, monitoring with a quartz microbalance. The C_60_ coverage was calibrated from 2PPE measurements (Supplementary Figs S1 and S2). TR-2PPE measurements were carried out using the third harmonics (*hν* = 4.13–4.77 eV, *p*-polarization) of a Ti:sapphire laser (~100 fs, 76 MHz), which was separated into pump and probe photons with a beam splitter. Both pump and probe photons are recombined at the sample in UHV with a concave mirror (*f* = 400 mm). In this optical configuration (“skew configuration”) the autocorrelation of incident photons can approximate a Gaussian peak function without interference oscilation^31^. Photoelectrons emitted in the normal direction were detected by a hemispherical electron energy analyzer (VGSCIENTA: R3000), where the energy of photoelectron was calibrated by UPS of an Au plate providing a clear Fermi edge as zero binding energy at *E*_final_ = *E*_F_ + *hν* (21.22 eV) for *E*_final_ (Supplementary Fig. S5). A sample bias of −3 V was applied to collect low-energy photoelectrons around the cut-off energy (*E*_vac_). Energy and time resolutions of the TR-2PPE system were about 20 meV and 20 fs, respectively. The sample temperature was 293 K during the TR-2PPE measurement.

## Additional Information

**How to cite this article**: Shibuta, M. *et al*. Direct observation of photocarrier electron dynamics in C_60_ films on graphite by time-resolved two-photon photoemission. *Sci. Rep.* **6**, 35853; doi: 10.1038/srep35853 (2016).

## Supplementary Material

Supplementary Information
