# Supplementary material for: Direct observation of photocarrier electron dynamics in C60 films on graphite by time-resolved two-photon photoemission
Source: Sci Rep. 2016 Oct 24;6:35853. doi: 10.1038/srep35853 (PMC5075791; doi:10.1038/srep35853)
Supplement: Supplementary Information [file srep35853-s1.doc]

***Supplementary Information***

**Direct observation of photocarrier electron dynamics in C_60_ films on graphite by time-resolved two-photon photoemission**

Masahiro Shibuta^1^, Kazuo Yamamoto^2^, Tsutomu Ohta^2^, Masato Nakaya^2,3^, Toyoaki Eguchi^2,3^ & Atsushi Nakajima^1,2,3,*^

^1^ *Keio Institute of Pure and Applied Science (KiPAS), Keio University, 3-14-1 Hiyoshi, Kohoku-ku, Yokohama 223-8522, Japan*

^2^ *Department of Chemistry, Faculty of Science and Technology, Keio University, 3-14-1 Hiyoshi, Kohoku-ku, Yokohama 223-8522, Japan*

^3^ *JST, ERATO, Nakajima Designer Nanocluster Assembly Project, 3-2-1 Sakado, Takatsu-ku, Kawasaki, 213-0012, Japan*

*Addresses correspondence to

Tel: +81-45-566-1712

Fax: +81-45-566-1697

E-mail: nakajima@chem.keio.ac.jp

**Calibration of C_60_ coverage.** The C_60_ coverage was calibrated from 2PPE measurements, while the deposition rate was monitored by a quartz microbalance. Supplementary Figure S1 shows the coverage dependence of 2PPE spectra for C_60_ on HOPG. The C_60_ deposition times are indicated on the right hand in the figure. For the clean HOPG substrate, a sharp peak is observed at *E*_F_ + 3.6 eV, which has been attributed to the first image potential state (IPS_HOPG_) formed on the clean HOPG surface^1^. The work function (*E*_vac_ – *E*_F_) determined from the low-energy cutoff of the spectra is 4.47 eV for the clean HOPG surface. The IPS_HOPG_ intensity and the work function of the surface generally sensitively change with the coverage of adsorbate up to 1 ML. Supplementary Figure S2 shows the change in the IPS_HOPG_ intensity and workfunction as a function of the deposition time. The changes were almost saturated at the deposition time of 14 min, showing that a fully covered 1 ML film had been achieved. Note that the spectral feature at 1 ML (14 min deposition) is slightly different from that taken with the corresponding photon energy in Fig. 1a, because the spectra in Fig. S1 are taken with a wider acceptance angle of ±5°.

**Supplementary Figure S1**. **C_60_ deposition time dependence of 2PPE.** Spectra are taken at a photon energy of 4.33 eV.

**Supplementary Figure S2**. **Changes in workfunction and IPS intensity versus C_60_ deposition time.** 1 ML is defined as 14 min deposition.

**Angle-resolved 2PPE; Energy dispersion of F0-F2.** In the main text, the observed energy levels of the F0–F2 states located above *E*_vac_ are assignable to a series of superatomic molecular orbitals (SAMOs) of C_60_ because of their highly dispersive character parallel to the surface. Here, supplementary Fig. S3 shows second derivative angle-resolved 2PPE for a 1 ML C_60_ film on HOPG with a photon energy of 4.33 eV. The horizontal and vertical axes represent the photoelectron kinetic energy and photoemission angle, respectively. The top spectrum is the 2PPE spectrum integrated over all the emission angles. The data clearly indicate that the electronic states of F0–F2 exhibit large band dispersions parallel to the surface, whereas the L0 (LUMO) and L1 (LUMO+1) show little dispersion. Also, it seems that the F0–F2 peaks are enhanced by resonance photoexcitation with L1, implying that an appropriate electronic state for resonance is required to observe the SAMOs with 2PPE.

**Supplementary Figure S3**. **Angle-resolved 2PPE**. Second derivative angle-resolved 2PPE for 1 ML C_60_ film on HOPG with a photon energy of 4.33 eV. Note, a −3 V sample bias was applied during the measurement.

**Coverage dependence of 2PPE.** Supplementary Figure S4 shows the C_60_ coverage dependence (0–30 ML) of 2PPE spectra taken with *hν* = 4.33 eV. Although the electronic structure is significantly perturbed on metallic substrates^2,3^, the energy of the LUMO is almost independent of the C_60_ coverage on HOPG owing to the distinct character of the 2D substrate of HOPG.

**Supplementary Figure S4**. **Coverage dependence of 2PPE spectra for C_60_ film on HOPG.**

**UPS spectrum for C_60_/HOPG and energy calibration of the spectrometer.** A conventional UPS spectrum for 1 ML C_60_/HOPG obtained by using He-Iα resonance line (*hν* = 21.22 eV) is shown in Supplementary Fig. S5. The C_60_-derived HOMO (2.2 eV) and HOMO–1 (3.6 eV) levels are clearly observed, which is consistent with previous reports^4,5^. The band edge of the C_60_ film (onset of HOMO) is 1.8 eV. A very weak structure just below *E*_F_ is a trivial artifact due to photoemission from the HOMO with a satellite resonance of the He-Iβ resonance line (*hν* = 23.09 eV). The upper right spectrum is that for an Au substrate measured at 90 K to calibrate the energy of photoelectrons in the spectrometer. This calibrated energy is also used in the 2PPE measurements in this study.

**Supplementary Figure S5. UPS spectrum for 1 ML C_60_ / HOPG.**

**Thickness dependence of LUMO+1 lifetime.** Supplementary Figure S6 shows intensity trace of LUMO+1 for the C_60_ thicknesses of 1, 2, 3, and 5 ML. Independent of the thickness, the LUMO+1 lifetime seems short within the time resolution at that time (~100 fs).

**Supplementary Figure S6. Intensity of LUMO+1 against Δ*t* for 1, 2, 3, and 5 ML C_60_.**

**Surface structure of C_60_/HOPG.**  Supplementary Figure S7a shows an STM image of ~1.4 ML (200 × 200 nm^2^) C_60_ on HOPG. Individual C_60_ molecules are recognized in the magnified image in the monolayer region as shown in S7b (50 × 50 nm^2^). A molecularly-ordered monolayer and second layer are formed in large areas, showing layer-by-layer growth of C_60_ at the low coverage. At a high coverage of ~4 ML (Fig. S7c), however, from three to five layers of C_60_ layer coexist on the surface, where no layer-by-layer growth mode is found.

**Supplementary Figure S7**. **STM images of C60 film on HOPG**. STM images of C_60_ / HOPG for the coverages of (a) ~ 1.4 ML (200 × 200 nm^2^), (b) ~1.4 ML magnified at monolayer region (25 × 25 nm^2^), and ~4 ML (200 × 200 nm^2^). The tip bias and tunneling current were −2 V, 20 pA for (a), −1.5 V, 20 pA for (b), and −2 V, 20 pA for (c), respectively.

**References**

1. Shibuta, M., Yamamoto, K., Miyakubo, K., Yamada, T. & Munakata, T. Resonant effects on two-photon photoemission spectroscopy: Linewidths and intensities of occupied and unoccupied features for lead phthalocyanine films on graphite. *Phys. Rev. B* **81**, 115426 (2010).

2. Dutton, G. Quinn, D. P., Lindstrom, C. D. & Zhu, X.-Y. Exciton dynamics at molecule-metal interface: C_60_/Au(111). *Phys. Rev. B* **72**, 045441 (2005).

3. Dutton, G. J., Dougherty, D. B., Jin, W., Reutt-Robey, J. E. & Robey, S. W. Superatom orbitals of C_60_ on Ag(111): Two-photon photoemission and scanning tunneling spectroscopy. *Phys. Rev. B* **84**, 195435 (2011).

4. Lof, R. W., van Veenendaal, M. A., Koopmans, B., Jonkman, H. T. & Sawatzky, G. A. Band gap, excitons, and Coulomb interaction in solid C_60_, *Phys. Rev. Lett*. **68**, 3924−3927 (1992).

5. Rudolf, P., Golden, M. S. & Brühwiler, P. A. Studies of fullerenes by the excitation, emission, and scattering of electrons. *J. Elect. Spectrosc. Relat. Phenom*. **100**, 409−433 (1999).
